# Short-Term High- and Moderate-Intensity Training Modifies Inflammatory and Metabolic Factors in Response to Acute Exercise

**DOI:** 10.3389/fphys.2017.00856

**Published:** 2017-10-31

**Authors:** Fabio Santos Lira, Thaislaine dos Santos, Renan Santos Caldeira, Daniela S. Inoue, Valéria L. G. Panissa, Carolina Cabral-Santos, Eduardo Z. Campos, Bruno Rodrigues, Paula A. Monteiro

**Affiliations:** ^1^Exercise and Immunometabolism Research Group, Department of Physical Education, State University (Unesp), School of Technology and Sciences, Presidente Prudente, São Paulo, Brazil; ^2^Department of Sport, School of Physical Education and Sport, University of São Paulo, São Paulo, Brazil; ^3^Department of Physical Education, Federal University of Pernambuco, Recife, Brazil; ^4^Faculty of Physical Education, University of Campinas, Campinas, Brazil

**Keywords:** exercise, metabolism, cytokines, IL-6, TNF-α

## Abstract

**Purpose:** To compare the acute and chronic effects of high intensity intermittent training (HIIT) and steady state training (SST) on the metabolic profile and inflammatory response in physically active men.

**Methods:** Thirty recreationally active men were randomly allocated to a control group (*n* = 10), HIIT group (*n* = 10), or SST group (*n* = 10). For 5 weeks, three times per week, subjects performed HIIT (5 km 1-min at 100% of maximal aerobic speed interspersed by 1-min passive recovery) or SST (5 km at 70% of maximal aerobic speed) while the control group did not perform training. Blood samples were collected at fasting (~12 h), pre-exercise, immediately post, and 60 min post-acute exercise session (pre- and post-5 weeks training). Blood samples were analyzed for glucose, non-ester fatty acid (NEFA), and cytokine (IL-6, IL-10, and TNF-α) levels through a three-way analysis (group, period, and moment of measurement) with repeated measures in the second and third factors.

**Results:** The results showed an effect of moment of measurement (acute session) with greater values to TNF-α and glucose immediately post the exercise when compared to pre exercise session, independently of group or training period. For IL-6 there was an interaction effect for group and moment of measurement (acute session) the increase occurred immediately post-exercise session and post-60 min in the HIIT group while in the SST the increase was observed only 60 min post, independently of training period. For IL-10, there was an interaction for training period (pre- and post-training) and moment of measurement (acute session), in which in pre-training, pre-exercise values were lower than immediately and 60 min post-exercise, in post-training period pre-exercise values were lower than immediately post-exercise and immediately post-exercise lower than 60 min post, it was also observed that values immediately post-exercise were lower pre- than post-training, being all results independently of intensity (group).

**Conclusion:** Our main result point to an interaction (acute and chronic) for IL-10 showing attenuation post-training period independent of exercise intensity.

## Introduction

The benefits of an active lifestyle are well-known, since regular practice of exercise imposes a series of challenges on bioenergetic pathways and active skeletal musculature, resulting in metabolic adaptations (Rivera-Brown and Frontera, 2012). Furthermore, physical exercise promotes increases in the immunological function principally through anti-inflammatory response, mediated by cytokines (Pedersen, 2009; Neto et al., 2011). These modifications depend on fundamental aspects of the training such as intensity, duration, and session volume (Pedersen, 2009; Neto et al., 2011; Lira et al., 2012).

Studies have evidenced the efficiency of endurance training programs, promoting fat loss, and improving aerobic capacity and cardiorespiratory benefits, among others (Sigal et al., 2014; Huang et al., 2016). However, more recently, studies have shown that high-intensity intermittent training (HIIT) also leads to similar, or even higher improvement in the same variables when compared with steady state training (SST) (Robinson et al., 2015; Franchini et al., 2016; Gerosa-Neto et al., 2016; Panissa et al., 2016).

Recently, we (Cabral-Santos et al., 2015, 2016a,b; Lira et al., 2015; Inoue et al., 2016) and others (Cullen et al., 2016; Dorneles et al., 2016; Wadley et al., 2016) have shown that a single bout of high-intensity intermittent exercise (HIIE) as well as steady state exercise (SSE) are effective for improving glucose tolerance, promoting an antiatherogenic response by increasing adiponectin and brain-derived neurotrophic factor (BDNF), and altering cytokine response leading to an anti-inflammatory status, however these modifications are dependent on exercise protocol, body fat (lean or obese subjects), and physical fitness levels (Cabral-Santos et al., 2015; Lira et al., 2015; Dorneles et al., 2016; Inoue et al., 2016).

Cytokines exert several functions that act on different cell types and have a crucial role in energy metabolism (Pedersen and Febbraio, 2008). For example, muscle contraction leads to activation of the c-Jun N-terminal kinase (JNK/AP-1) and mitogen-activated protein kinase (MAPK) in muscle cells that raises Interleukin 6 (IL-6) and Tumor necrosis factor alpha (TNF-α) levels immediately in response to acute aerobic exercise and act as a cross-talk between skeletal muscle and immune cells (Pal et al., 2014). They have been considered energetic sensors capable of signaling in a hormone-like manner to mobilize extracellular glucose and induce pronounced lipolysis during exercise (Febbraio and Pedersen, 2005; Kim et al., 2015).

The increase in IL-6 is closely related to the muscle mass involved in contractile activity, exercise modalities that involve a large number of muscle groups present more pronounced increases in IL-6 (Pedersen and Febbraio, 2008), in addition to which, the exercise intensity also plays a role in the magnitude of this response, with high intensity exercises leading to a greater increase in IL-6 post-exercise (Cabral-Santos et al., 2015). In addition, Interleukin-10 (IL-10) and Interleukin 1 receptor antagonist (IL-1ra) levels increase in response to exercise, and their suggested function is to prevent exacerbation of the pro-inflammatory response (Lira et al., 2015). Interleukin 10 (IL-10) increases in response to HIIE in a similar manner to SSE, when session volume is matched (Cabral-Santos et al., 2015). Although acute responses are known it is important to investigate if these acute modifications change chronically. This observation can be made in a fasted state as in the majority of studies; however, it is also important to verify acute response to exercise after a training period (Zwetsloot et al., 2014; Monteiro et al., 2017).

In this context, the aims of the present study were to analyze the effects of 5 weeks of HIIT or SST on energetic molecules (glucose and non-ester fatty acid levels), and systemic cytokine parameters (IL-6, IL-10, and TNF-α levels) in an acute exercise bout performed before and after the training period.

## Materials and methods

### Subjects

Men, non-obese and physically active (BMI ≤ 25; WHO, 2000), were invited to participate in the study through divulgation of the project in social networks, printed posters, and email lists of students and employees at the Universidade Estadual Paulista—Campus Presidente Prudente. Thirty subjects (age 26.36 ± 4.19 years, weight 74.37 ± 9.26 kg, height 1.77 ± 0.06 m, and peak oxygen uptake 52.82 ± 4.96 mlkg^−1^ min^−1^) were enrolled for the present study. The participants presented a health and neuromuscular status that ensured their ability to complete the study protocol. Written informed consent was obtained from all subjects after they had been informed about the purpose and risks of the study. All procedures of this study were approved by the Research Ethics Committee for studies involving human participants of the State University (Unesp), School of Technology and Sciences, Presidente Prudente/SP (53297815.8.0000.5402).

Our primary hypothesis was that changes in inflammatory markers in the SST and HIIT groups of men after 5 weeks of training would be statistically significant, with a power (1-type II error) of 0.80 and a type I error of 0.05 based on IL-10. For this hypothesis, we used a study that measured differences between both protocols (Wadley et al., 2015) and studies that measured the IL-6 pre and immediately post-exercise as referenced by a similar protocol (high-intensity intermittent exercise) (Meckel et al., 2009, 2011; Leggate et al., 2010; Lira et al., 2015). Before conducting the study we verified the sample size needed (*n* = 6) using G^*^Power 3.1 software (Düsseldorf, Germany).

### Study design

**Figure 1:**
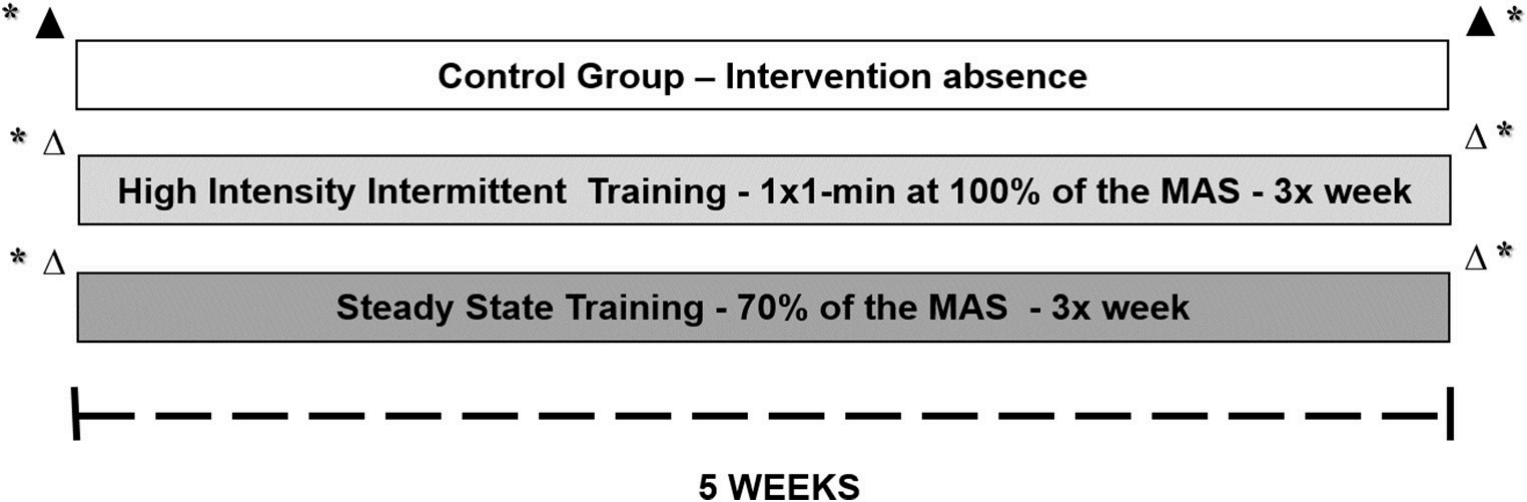
Schematic representation of protocols and study design. Δ Acute session. ^*^Evaluation for characterization of subjects and Incremental test (MAS). ▲ Only blood sample collection.

**Figure 2:**
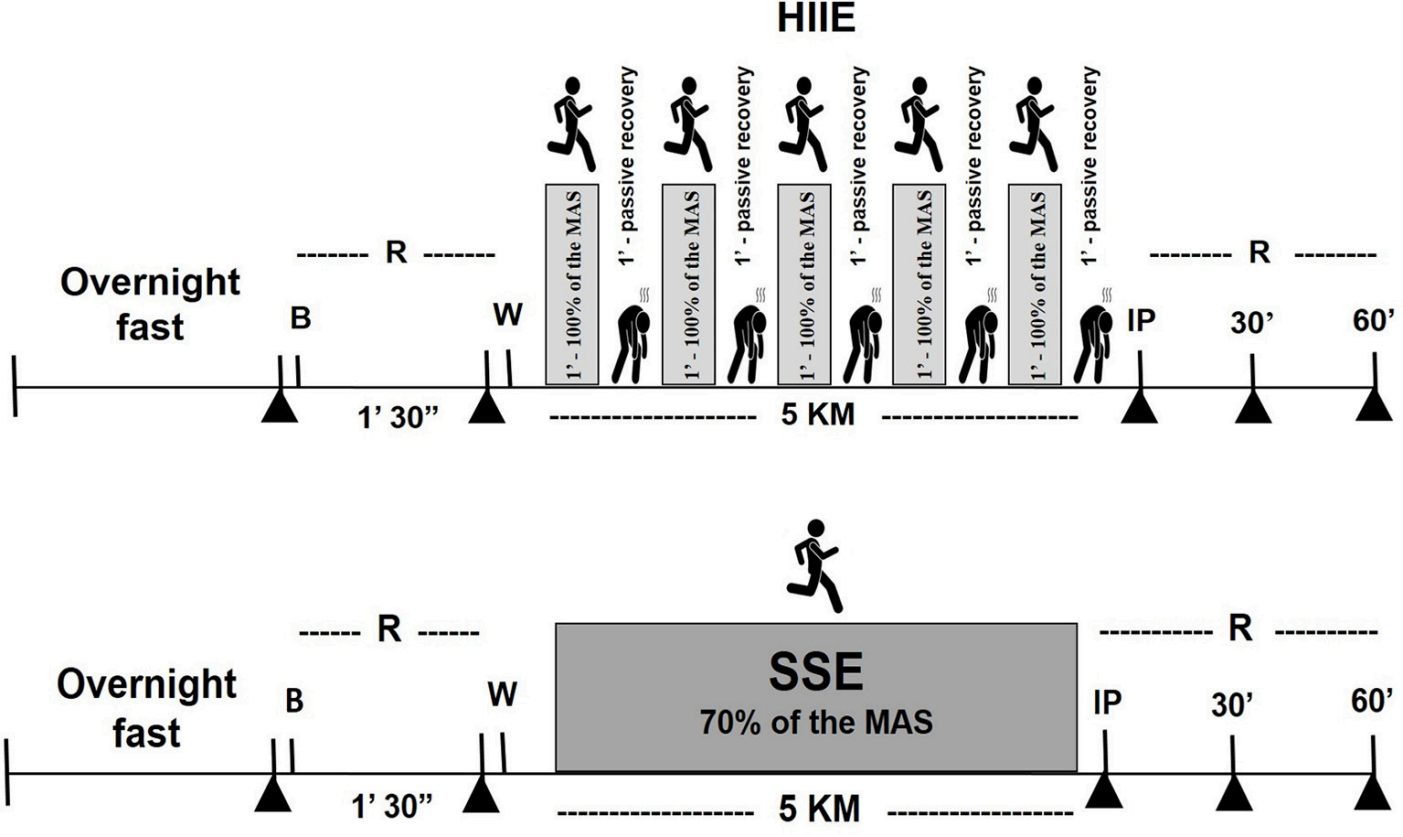
Schematic representation of the acute session of the study protocol, steady-state exercise (SSE) or high-intensity intermittent exercise (HIIE). ▲ Collection of blood samples at overnight fasting, pre exercise, immediately, and 30 and 60 min after exercise). R, rest; B, breakfast; W, warm up; IP, immediately post-exercise.

Posteriorly, subjects was stratified into three groups: HIIT (*N* = 10–exercised 1:1–1 min of running at 100% of velocity correspondent to maximal aerobic speed (MAS) and 1 min of passive recovery, until completing a total volume of 5-km per session); SST (*N* = 10–exercised continuously at 70% of MAS, completing a total volume of 5-km per session); and control group (CG) (*N* = 10–continued their training routine, two university football players, five individuals who participated in a local CrossFit group, one amateur jiu-jitsu practitioner, and two military men who performed regular physical exercises). The groups underwent 5 weeks of aerobic training with a frequency of three times a week on a treadmill, except for the CG, which performed no intervention. The participants were submitted to an incremental test and anthropometry. Blood was collected from the participants in two acute session, in the first and last training session. All evaluations pre intervention were repeated in identical conditions after 5 weeks. Figures 1, 2 present these acute and chronic evaluations.

### Incremental test for determination of maximum speed and peak oxygen consumption

The subjects were submitted to an incremental test for determination of aerobic fitness on the treadmill (Inbramed, model MASTER CI, Brazil), with the measurement of maximum oxygen consumption (Model Quark PFT Ergo, Cosmed, Rome), until voluntary exhaustion (see Cabral-Santos et al., 2015). The initial speed was set at 8 km h^−1^ with an increase of 1 km h^−1^ every 2 min. The MAS was assumed as the final completed stage. If the subjects stopped before the end of the stage, the MAS was determined according to Kuipers et al. (1985).

### Experimental protocols

#### High-intensity intermittent training

Subjects performed a 5 km run intermittently; being 1-min at MAS followed by 1-min of passive recovery (the subjects remained standing or sitting after each exercise bout). The general warm-up was performed at 50% of MAS for 5 min. Subjects performed training three times per week on non-consecutive days.

#### Steady state training

Subjects performed a 5 km run continuously at 70% of MAS (determined in the incremental test) on the treadmill. The general warm-up was performed at 50% of maximum speed for 5 min. Subjects performed training three times per week on non-consecutive days.

### Acute session

The volunteers were randomly divided into two groups (HIIE or SSE) and performed a controlled acute session on the first (pre-training) and last (post-training) exercise day of the 5-week training period (chronic effect). On the day of the acute sessions all volunteers performed the first fasting blood collection (8–12), then ingested a standard breakfast (consisting of yogurt, toast, and cottage cheese) with energy value stipulated according to body composition (25% of daily energy needs), comprising energy values distributed between carbohydrates (52%), lipids (35%), and proteins (13%). After breakfast, the volunteers remained 1 h at rest, and then the second blood collection occurred. After the second blood collection, the volunteers began the acute training session. Exactly after the training session there was a new blood collect, as well as 30 (analyzed only IL-10) and 60 min after the end of the exercise session (acute effect).

### Blood samples

The blood samples (15 ml) were immediately allocated into two 5 ml vacutainer tubes (Becton Dickinson, BD, Juiz de Fora, MG, Brazil) containing EDTA for plasma separation and into one 5 ml dry vacutainer tube for serum separation. The blood was centrifuged at 3,000 rpm for 15 min at 4°C. Serum and plasma were then stored in Eppendorf plastic tubes and stored at −20°C for future analysis.

#### Blood sampling and analyses

The concentrations of IL-6 (cod. S6050.) (Sensitivity 0.7 pg/ml; Assay Range 3.1–300 pg/ml), IL-10 (cod. S1000B) (Sensitivity 3.9 pg/ml; Assay Range 7.8–500 pg/ml), and TNF-α (cod. STA00C) (Sensitivity 5.5 pg/ml; Assay Range 15.6–1,000 pg/ml) were analyzed by ELISA commercial kits (R&D Systems, 614 McKinley Place NE, Minneapolis, MN 55413, USA). Glucose was assessed using commercial kits (Labtest®, São Paulo, Brazil). Non-ester fatty acid (NEFA) was assessed by a colorimetric method with a commercial kit (Wako, 1-2, doshomachi 3-Chome, Chuo-Ku, Osaka 540-8605, Japan).

### Statistical analysis

Data normality was verified using the Shapiro-Wilk test and descriptive data are shown as means and standard deviation. Two-way analysis of variance (ANOVA) with repeated measures was used to compare the differences in metabolic variables and inflammatory markers between groups (control, HIIT, and SST) at baseline (fasting) and training period (pre- and post-5 weeks of exercise). Three-way analysis of variance (ANOVA) with repeated measures was applied to compare the inflammatory and metabolic response to acute exercise session according to group (HIIT and SST), training period (pre- and post-5 weeks), and moment of measurement of the collection of the blood samples in acute session (at rest, immediately- and 60-min post-exercise). Statistical significance was set at 5% for all analysis and the calculations were conducted using SPSS, version 17.0 (SPSS Inc. Chicago. IL).

## Results

Table 1 presents the comparison between baseline values (metabolic variables and inflammatory markers) of the volunteers, pre- and post-5 weeks of HIIT and SST, as well as the control group. In this table that considered just the fasted values pre- and post-training, there was an main effect for group for glucose (*F* = 5.29; *p* = 0.012; partial η^2^ = 0.282); with the values of the control group being greater than HIIT and SST (*p* = 0.018; *p* = 0.042, respectively).

**Table 1:** Metabolic and inflammatory responses at fasting, pre- and post-training in control, steady state and high intensity intermittent training groups.

		**Pre-training**	**Post-training**
IL-6 (pg·ml^−1^)	Control	1.47(0.410)	1.44(0.581)
	SST	1.36(0.784)	1.52(0.807)
	HIIT	1.09(0.551)	1.24(0.862)
IL-10 (pg·ml^−1^)	Control	5.41(2.67)	3.39(1.97)
	SST	3.39(2.02)	3.33(1.82)
	HIIT	4.66(1.30)	3.29(1.81)
TNF- α (pg·ml^−1^)	Control	2.98(0.469)	4.13(1.007)
	SST	3.63(0.646)	2.83(1.728)
	HIIT	3.28(0.518)	4.26(0.931)
IL-6/IL-10	Control	0.24(0.073)	0.67(0.534)
	SST	1.37(2.041)	0.50(0.290)
	HIIT	0.23(0.155)	0.323(0.176)
NEFA (mmol/l)	Control	0.93(0.058)	0.90(0.040)
	SST	0.94(0.093)	0.89(0.074)
	HIIT	0.93(0.089)	0.94(0.059)
Glucose (mg/dl)	Control	86.58(5.932)	92.08(5.298)^*^
	SST	80.89(8.843)	79.91(10.353)
	HIIT	78.00(5.975)	80.43(10.946)

*Values are mean ± standard deviation*.

* *, different of the other groups, p < 0.05. IL-6, Interleukin 6; IL-10, Interleukin 10; TNF- α, Tumor necrosis factor α; NEFA, non-ester fatty acid*.

Table 2 presents the values of IL-6, TNF-α, IL-10 and glucose in acute exercise sessions performed pre- and post-5 weeks of training performed in different intensities. All values were presented besides the values grouped by main effect (groups or training period) to show the differences more clearly.

**Table 2:** Metabolic and inflammatory responses at rest, immediately and 60-min post an exercise session in different intensities and pre- and post-5 weeks of training.

**Variables**		**Pre-training**		**Post-training**		**Intensity grouped**		**Training period grouped**	
		**HIIE**	**SSE**	**HIIE**	**SSE**	**Pre training**	**Post training**	**HIIE**	**SSE**
IL-6 (pg·ml^−1^)	Pre	1.29(0.56)	1.46(0.55)	1.25(0.50)	1.39(0.54)	1.37(0.55)	1.28(0.50)	1.27(0.51)	1.39(0.52)
	Post	2.93(1.59)	2.38(0.69)	2.10(0.78)	2.62(1.22)	2.65(1.23)	1.90(0.80)	2.51(1.29)^&^	2.04(0.81)
	60 min	2.21(0.83)	2.62(1.45)	1.90(0.76)	2.29(1.23)	2.41(1.17)	2.33(1.48)	2.05(0.79)^&^	2.69(1.65)^&^
TNF-α (pg·ml^−1^)	Pre^*^	2.56(0.66)	2.56(0.62)	2.47(1.02)	3.12(1.66)	2.56(0.62)	2.79(1.38)	2.51(1.75)	2.84(1.08)
	Post	3.80(2.12)	3.55(1.29)	3.17(1.52)	3.02(1.35)	3.67(1.71)	3.09(1.40)	3.48(1.35)	3.28(1.27)
	60 min	3.35(1.23)	3.61(1.22)	2.92(1.60)	2.50(0.90)	3.48(1.200)	2.71(1.28)	3.13(1.32)	3.05(1.15)
IL-10 (pg·ml^−1^)	Pre	4.12(1.86)	4.27(3.00)	3.11(1.73)	2.87(1.92)	4.20(2.40)^#^	2.99(1.76)^#^	3.61(1.80)	3.57(2.52)
	Post	7.67(2.58)	7.56(2.98)	3.63(1.29)	4.37(1.23)	7.62(2.68)^£^	4.00(1.27)^#^	5.65(2.87)	5.97(2.74)
	60 min	7.97(2.90)	7.54(4.41)	10.04(5.39)	5.76(4.26)	7.76(3.59)	7.90(5.27)	9.01(429)	6.65(4.26)
NEFA (mmol/l)	Pre	0.91(0.07)	0.901(0.07)	0.88(0.02)	0.91(0.07)	0.88(0.04)	0.86(0.04)	0.90(0.05)	0.89(0.06)
	Post	0.78(0.39)	0.82(0.30)	0.92(0.04)	0.93(0.07)	0.84(0.30)	0.93(0.08)	0.89(0.22)	0.87(0.23)
	60 min	0.96(0.08)	0.93(0.07)	0.92(0.05)	0.85(0.22)	0.95(0.07)	0.89(0.23)	0.94(0.06)	0.89(0.10)
Glucose (mg/dl)	Pre^*^	77.04(12.12)	82.56(9.57)	75.05(8.97)	79.27(9.97)	79.80(11.00)	77.16(9.48)	76.04(10.16)	80.9(9.42)
	Post	89.16(6.70)	80.86(10.59)	84.01(9.40)	85.95(8.94)	85.01(9.62)	84.98(8.99)	86.58(8.17)	83.40(9.65)
	60 min	81.04(16.23)	80.59(8.71)	76.55(8.14)	81.11(12.31)	80.82(12.68)	78.83(10.42)	78.79(12.39)	80.85(10.12)

*Values are mean ± standard deviation. All values were presented besides the values grouped by main effect (groups or training period) to show the differences more clearly*.

* , main effect for moment, different from immediately post-exercise (p < 0.05);

# *, interaction between group and moment, different from post-60 min (p < 0.05)*,

£ , different from post training at the same moment of measurement (p < 0.05);

& *, interaction effect for group and moment of measurement, different from pre-exercise for the same group (p < 0.05)*.

For IL-6 there was an interaction effect for group and moment of measurement [*F*_(2, 36)_ = 4.55; *p* = 0.017; partial η^2^ = 0.201], in the HIIT group pre-exercise values were lower than immediately- and 60 min post-exercise (*p* < 0.001; *p* = 0.036; respectively), in SST group pre-exercise values were lower than 60 min post-exercise (*p* < 0.001).

For TNF-α there was only a main effect for moment of measurement [*F*_(2, 36)_ = 6.19; *p* = 0.004; partial η^2^ = 0.256], being that pre-exercise values were lower than immediately post-exercise (*p* = 0.003).

For IL-10, there was an interaction for training period and moment of measurement[*F*_(2, 24)_ = 5.55; *p* = 0.010; η^2^ = 0.316], in which in pre-training period pre-exercise values were lower than immediately—(*p* = 0.003) and 60 min post-exercise (*p* = 0.002). In addition, in post-training period pre-exercise values were lower than immediately post-exercise (*p* < 0.001) and immediately post-exercise lower than 60 min post-exercise (*p* < 0.001). It was also observed that values immediately post-exercise were lower pre- than post-training (*p* = 0.002), without differences for the other moments of measurement (pre- and post-60 min of exercise).

There was no effect for IL6/IL10 ratio or NEFA, but for glucose there was a main effect of moment of measurement [*F*_(2, 36)_ = 4.68; *p* = 0.015; η^2^ = 0.206], being that pre-exercise was lower than immediately post-exercise (*p* = 0.017).

## Discussion

The aims of the present study were to analyze the acute and chronic effects of HIIT or SST on metabolic profile and systemic cytokine parameters. The main findings of the present study were that (i) HIIT exerted more impact on IL-6 response in the acute exercise session independent of training period since IL-6 increased in the acute HIIT and SST sessions but after the HIIE protocols this increase occurred immediately and 60 min post-exercise while after an SSE session IL-6 increased only 60-min after the exercise session; (ii) TNF-α increased immediately post-acute exercise session independent of intensity and training period, and (iii) finally, IL-10 increased immediately after an acute exercise session independent of training period and intensity, however this increase was less post-training compared with pre-training, showing an attenuation of this increase. To the best of our knowledge, this is the first study to examine acute and chronic metabolic and inflammatory responses to SST and HIIT in physically active young men.

In the present study no effect of intensity was found on metabolic and inflammatory parameters fasting or after a training period. Moreover, another factor that can modulate the immunological and metabolic response to exercise is the pleiotropic IL-6. Only during contractile activity, muscle *per se* produces and releases IL-6 in several folds in a duration dependent manner (Pedersen, 2009). IL-6 showed a moment of measurement effect with a peak immediately after an HIIE session, and a delay in peak after an SSE session (1 h after acute exercise), showing that the HIIT group presented more effect in acute IL6-response. This finding can be related, at least in part, to a reduction in intramuscular glycogen availability which favors activations in the pathway involved in the production of IL-6 (Pedersen, 2009). Studies have observed that IL-6 increases in skeletal muscle, liver, and adipose tissue by 30–150%, in an animal model accompanied by high activity of AMPK in IL-6 production due to muscle contraction. Infusion IL-6 in males recreationally physically active showed that treatment with recombinant (rhIL-6) in concentrations similar to exercise ameliorate the glucose metabolism, being able to elevate GLUT4 translocation and consequently increase the availability of insulin-stimulated glucose (Kelly et al., 2004; Carey et al., 2006).

There was also an acute effect of time of measurement for TNF-α, with higher values immediately post-exercise than pre-exercise independent of the group (intensity). Consistent with previous reports, a time effect in plasma production of TNF-α was observed in both groups with higher values immediately after the exercise session. This increase suggests, at least in part, a possible lipolysis process in favor of increasing the availability of fatty acids into the blood circulation from adjacent tissues (Pedersen, 2009; Cabral-Santos et al., 2015), in order to maintain contractile activity due to the characteristics of the exercise. However, if prolonged, the elevated TNF-α level can be deleterious, even leading to insulin resistance by downregulating the tyrosine kinase activity of the insulin receptor (Pedersen, 2009). The mechanisms to counteract this mechanism have been assigned to an even more exacerbated increase in IL-10 concentration to attenuate possible deleterious effects, such as the activity of the transcriptional factor NF-κB pathway in target genes related to several pro-inflammatory cytokines including TNF-α, IL-1α, and IL-1β (Pedersen, 2009; Cabral-Santos et al., 2015).

At lower/moderate intensities and prolonged durations of exercise (45–60 min of exercise session at 65–75% of VO_2max_) the high aerobic energy demand depletes glucose rate and promotes a “metabolic shift” in the contributions of fuel (Jeppesen and Kiens, 2012). In our study, the concentration of glucose increased after the sessions in both group's pre- and post-training, although no significant changes were observed in NEFA. Improvements in performance can be achieved through training at or near VO_2max_ (Buchheit and Laursen, 2013) and there is an adaptation to the training favoring the aerobic metabolism through improvement in free fatty acid uptake and its oxidation in skeletal muscle. However, 5 weeks of HIIT seems not be enough to provide sufficient stimulus to improve this parameter.

Finally, the increase in IL-10 concentration immediately after the acute exercise session pre-training (both HIIE and SSE) was attenuated in the acute exercise session post-training, demonstrating that short-term aerobic training (5-weeks), independent of the intensity and type (moderate-intensity continuous or high-intensity intermittent), leads to adaptation in anti-inflammatory pathways. Leggate et al. (2012), in a study with overweight and obese sedentary young men (18–34 years), during 2 weeks of HIIT on a cycle ergometer (4-min at ~90% of maximal heart rate, with 2 min recovery, three times a week), showed that HIIT is able to modulate IL-6 source of adipose tissue after only 2 weeks. Another study conducted by Zwetsloot et al. (2014), also evaluated the effects of 2 weeks of HIIT on a cycle ergometer (60 s of exercise, load corresponding to VO_2_max, with 75 s of active recovery, three times a week) on inflammatory response in eutrophic men, physically active. The authors found an acute session of HIIT induced significant increases in IL-6, IL-8, IL-10, TNF-α, and MCP-1 (monocyte chemotactic protein-1) compared with rest, however 2 weeks of HIIT did not change this inflammatory response.

IL-10 exerts function in different cell types and induces the suppression of the inflammatory response; its biological action is interceded by its membrane receptor (IL-10R). Therefore, IL-10 can inhibit the production of several cytokines such as IL-1β and TNF-α, which is transcriptionally controlled by NF-kB pathway. Therefore, the potential mechanisms about the effect of exercise training in diseases condition (e.g., Obesity, diabetes type 2, sedentary, and others), which modulates the production of TNF-α by increasing IL-10 (Teixeira et al., 2016).

To the best of our knowledge, this is the first study to examine the IL-10 responses in an acute exercise session pre and post-training (SST and HIIT). The decrease in plasma IL-10 concentration appears to be down-regulated by training and may characterize a normal adaptation. It is noteworthy that Keller et al., 2005) demonstrated that after a 10 weeks training period, the down-regulation of IL-6 is partially counteracted by enhanced expression of IL-6R, suggesting a sensitization of skeletal muscle to IL-6 at rest. Similarly, there is virtuous evidence that training programs result in a decrease in IL-10 levels chronically, however the mechanism involved needs be determined by further studies.

Overall, the advantage of our study is the exploration of the kinetics of inflammatory and metabolic profile during and after acute exercise (pre-training), as well as, chronic analysis of the acute effect of the exercise (post-training). On the other hand, the limitations were analyzing the inflammatory response in a eutrophic and physically active population, considering that this population is not affected by low-grade chronic inflammation, such as in sedentary individuals or those with diabetes, obesity and other diseases. The other point is the time of intervention, that as suggested by other studies may not be sufficient to promote significant changes in the metabolic and inflammatory profile. Thus, further studies with longer-lasting interventions deserve investigation.

Taken together, the present findings suggest that a similar adaptation may occur through cytokine release, independent of stimulus, observed in HIIT when compared to SST. The present study data support that submitting physically active young men to an exercise program is associated with beneficial effects on metabolic and inflammatory adaptations through exercise-induced cytokine release after 5 weeks.

## Author contributions

Study design and organization of the manuscript were performed by FS, TdS, RS, DI, VP, CC, EC, BR, and PM. Data analysis, statistical analysis, and the first draft of the manuscript were performed by FS, DI, VP, DI, BR, and PM. The manuscript review was performed by EC, PM, VP, DI, and FS. The final approval for publication was performed by FS.

### Conflict of interest statement

The authors declare that the research was conducted in the absence of any commercial or financial relationships that could be construed as a potential conflict of interest.

## Abbreviations

AMPK: Adenosine monophosphate-activated protein kinase
ANOVA: Analysis of variance
BDNF: Brain-derived neurotrophic factor
CG: Control group
GLUT4: Glucose transporter type 4
HIIE: High-intensity intermittent exercise
HIIT-: High intensity intermittent training
IL-10: Interleukin-10
IL-1ra: Interleukin 1 receptor antagonist
IL-1α: *Interleukin 1* alpha
IL-1β: *Interleukin 1* beta
IL-6: Interleukin 6
IL-6R: Interleukin 6 receptor
IL-8: Interleucina−8
JNK/AP-1- C-Jun: N-terminal kinase
MAPK: Mitogen-activated protein kinase
MAS: Maximal aerobic speed
MCP-1: Monocyte chemotactic protein-1
NEFA: Non-ester fatty acid
NF-κB: Nuclear transcription factor kappa B
rhIL-6: Recombinant of interleucina-6
SSE: Steady state exercise
SST: Steady state training
TNF-α: Tumor necrosis factor alpha
VO_2max_: Maximal oxygen uptake.
